# Co-Delivery of Letrozole and Cyclophosphamide via Folic Acid-Decorated Nanoniosomes for Breast Cancer Therapy: Synergic Effect, Augmentation of Cytotoxicity, and Apoptosis Gene Expression

**DOI:** 10.3390/ph15010006

**Published:** 2021-12-21

**Authors:** Hamidreza Sahrayi, Elham Hosseini, Sara Karimifard, Nazanin Khayam, Seyed Mohammadmahdi Meybodi, Sahar Amiri, Mahsa Bourbour, Bahareh Farasati Far, Iman Akbarzadeh, Mohammed Bhia, Clare Hoskins, Chaiyavat Chaiyasut

**Affiliations:** 1 Department of Chemical and Petrochemical Engineering, Sharif University of Technology, Tehran 1458889694, Iran; h.sahrayi72@alumni.um.ac.ir (H.S.); karimifard.sara@gmail.com (S.K.); nazaninkhayam@gmail.com (N.K.); saharamiri@gmail.com (S.A.); iman.akbarzadeh@che.sharif.edu (I.A.); 2 Department of Biomedical Engineering, Science and Research Branch, Islamic Azad University, Tehran 1477893855, Iran; elhamhosseini72@gmail.com; 3 Department of Veterinary Medicine, Tabriz Branch, Islamic Azad University, Tabriz 5157344533, Iran; smm.meibodi96@gmail.com; 4 Department of Biotechnology, Alzahra University, Tehran 1993893973, Iran; m.bourbour1@yahoo.com; 5 Department of Chemistry, Iran University of Science and Technology, Tehran 1684613114, Iran; 6 Student Research Committee, Department of Pharmaceutics and Nanotechnology, School of Pharmacy, Shahid Beheshti University of Medical Sciences, Tehran 1996835113, Iran; mohamadbahia1996@gmail.com; 7 Department of Pure and Applied Chemistry, University of Strathclyde, Technology Innovation Centre, 99 George Street, Glasgow G1 1RD, UK; clare.hoskins@strath.ac.uk; 8 Innovation Center for Holistic Health, Nutraceuticals, and Cosmeceuticals, Faculty of Pharmacy, Chiang Mai University, Chiang Mai 50200, Thailand

**Keywords:** breast cancer, neoplasms, niosomes, drug delivery, nanoparticles, nanomedicine, letrozole, cyclophosphamide, folic acid

## Abstract

Breast cancer is one of the most prevalent causes of cancer mortality in women. In order to increase patient prognosis and survival rates, new technologies are urgently required to deliver therapeutics in a more effective and efficient manner. Niosome nanoparticles have been recently employed as therapeutic platforms capable of loading and carrying drugs within their core for both mono and combination therapy. Here, niosome-based nanoscale carriers were investigated as a targeted delivery system for breast cancer therapy. The platform developed consists of niosomes loaded with letrozole and cyclophosphamide (NLC) and surface-functionalized with a folic-acid-targeting moiety (NLCPFA). Drug release from the formulated particles exhibited pH-sensitive properties in which the niosome showed low and high release in physiological and cancerous conditions, respectively. The results revealed a synergic effect in cytotoxicity by co-loading letrozole and cyclophosphamide with an efficacy increment in NLCPFA use in comparison with NLC. The NLCPFA resulted in the greatest drug internalization compared to the non-targeted formulation and the free drug. Additionally, downregulation of *cyclin-D*, *cyclin-E*, *MMP-2*, and *MMP-9* and upregulating the expression of *caspase-3* and *caspase-9* genes were observed more prominently in the nanoformulation (particularly for NLCPFA) compared to the free drug. This exciting data indicated that niosome-based nanocarriers containing letrozole and cyclophosphamide with controlled release could be a promising platform for drug delivery with potential in breast cancer therapy.

## 1. Introduction

Breast cancer is the most common type of cancer in women with millions of new cases detected annually. Once established, breast cancer metastasizes into bone, lung, and liver [1,2]. Due to the cancer cell metabolism’s complexity, there is no exact treatment method, but two common local treatments, such as surgery and radiotherapy, and systemic ones, such as chemotherapy and hormone therapy, are widely used [2,3]. After surgery, the metastasized cells can reform tumors after removal of the primary tumor, causing patient remission. Alternatives, such as radiotherapy [4,5] or chemotherapy, are highly non-specific to the cancerous tissue causing widespread tissue damage and harsh patient side effects. Additionally, the non-specificity of chemotherapy results in low delivery efficiency towards the site of need therefore requiring high-dose administration to reach the therapeutic window [4] or to overcome drug resistance, resulting in greater and more severe side effects [5].

Niosome nanoparticles are vesicles made from a nonionic bilayer structure that is formed via hydration in water with or without cholesterol or some other lipids [6,7] as stabilizing agents. Their vesicular structures are similar to liposomes and can be used as carriers for lipophilic or hydrophilic agents [8,9,10]. Niosomes offer an alternative approach to liposomes due to the fact of their lower toxicity, ease of fabrication, biocompatibility, structural flexibility, and low cost [11]. The niosomal bilayer allows for drug trafficking within the body, protecting the drug from premature degradation, and directing therapeutic payloads to their site of need, thus reducing the unwanted off-target effects, which result in detrimental patient side effects. Not only are niosomes biocompatible, but they are also biodegradable; they exist at the nanometer scale, representing a high potential choice for targeted cancer therapy [12].

Some cancer cell types possess positive estrogen and progesterone receptors, growing faster in estrogen and progesterone environments, respectively. These cells account for 60–65% of all breast cancer, and women are particularly at risk of developing these cancers post their first pregnancy or when in menopause [13,14]. Sadly, these cancers do not respond well to chemotherapy, and they are often are treated with estrogen and hormone therapy [13,15]. Hormone therapy is effective for the treatment of positive receptor cancers. It has been shown to directly result in tumor shrinkage. It is often preferred to chemotherapy due to the fact of its lower toxicity [16]. In hormone therapy, a novel generation of aromatase inhibitors (AIs) limit estrogen production [17]. Aminoglutethimide, as the first generation of AIs had some limitations such as cortisol and aldosterone synthesis. Steroid inhibitors, such as formestane, and non-steroidal inhibitors, such as fadrozole, as the second generation of AIs, possessed enhanced selectivity [17]. For improving fadrozole, Novartis derived the third generation of benzyl-azoles, such as letrozole (Let) from fadrozole, which possessed higher selectivity [18]. Antibody immunotherapy possessing high selectivity and low toxicity has shown great promise in cancer therapy; however, they exhibit low diffusivity into the bones and are ineffective against the metastasized cancer. In this case, hormone therapy and chemotherapy are needed [19,20,21]. Cyclophosphamide (Cyclo) is one of the oldest chemotherapies used to treat lung and breast cancer [22]. Moreover, it is applied to other types of cancer such as leukemia, lymph, bladder, and ovary [22]. Cyclo is an alkylation agent that can affect a different part of the immune system and result in synergistic effects when administered in combination with antibodies [23,24]. It can prevent DNA replication by DNA alkylation [22]. However, Cyclo use can also result in side effects, such as liver toxicity, cardiotoxicity, nephrotoxicity, bladder toxicity, cardiotoxicity, and anemia, which are all dependent on the dosage [22]. Cyclo has been reported as a chemotherapeutic agent alone or in combination with drugs such as capecitabine and methotrexate [25]. Reports have shown that Ais, such as letrozole (Let) co-administered with low dosages of Cyclo, result in better therapeutic outcomes compared to Let alone [16].

One of the most important outcomes when designing new drug delivery systems is the ability to produce site-specific, targeted, efficient platforms that enhance therapeutic effects over the current gold standards. One major advance in this area is in the co-loading of clinically used drugs to form a single platform combination therapy that is capable of producing a synergistic effect resulting in better therapeutic outcomes [26,27,28,29]. The use of two anticancer agents simultaneously that have two different mechanisms of actions can help in improving the therapeutic effects by covering more than one mechanism of action [30]. Therefore, there is a need to formulate these combination drug delivery systems with a wide range of anticancer drugs to find the optimal co-delivery strategy, such as the combination of Let (type II aromatase inhibitor) and Cyclo (alkylating agent).

In this study, different niosomal nanoformulations were fabricated via a thin-film method using cholesterol, Span 60, and folic acid as lipid, surfactant, and functionalization reagents. Let and Cyclo were co-loaded into the niosome nanoparticles and their ability to act in synergism resulting in enhanced cytotoxicity investigated on breast cancer cell lines. Varied surfactant:cholesterol and lipid:drug molar ratios were considered to optimize the physical and chemical characteristics of the formulations for further biological studies. In addition, to confer targeting abilities of the carriers, the nanoniosomes were coated with folic acid to interact with the folate receptors, which are upregulated in breast cancer cells. Finally, biological in vitro tests were performed including cell viability, apoptotic gene expression, and apoptosis ratios studied using MDA-MB-231 and SKBR3 as two different breast cancer cell lines.

## 2. Results and Discussion

### 2.1. Noisome Formulations Properties

Niosome nanoparticles containing an equal amount of Let and Cyclo drugs (10 mg each) at varied surfactant: cholesterol (1:1 and 2:1) and lipid: drug (10:1 and 20:1) molar ratios were prepared. The physical properties of the formulations, such as mean average nanoparticles sizes, particle size distributions, and EE% were analyzed and reported in Figure S1 and Table 1. The data showed that particle size of niosomes increased with the use of higher lipid-to-drug molar ratios. For instance, by using 20:1 molar ratio instead of 10:1 for lipid to drug, the particle size increased from 225 to 252 nm for niosomes that were prepared by using a 1:1 molar ratio of surfactant to cholesterol. In addition, a 231 to 241 nm size increment observed for niosomes that were prepared by using a 2:1 molar ratio in surfactant to cholesterol (*p* < 0.05), this was probably due to the fact of a thicker bilayer forming [31]. The EE% of the samples increased by 2–3% for each Let and Cyclo at the higher lipid: drug and surfactant: cholesterol molar ratios. It was proposed that the thicker bilayer formed was capable of a higher encapsulation capacity for the hydrophobic drugs [32,33,34]. The surfactant: cholesterol molar ratio significantly impacted the size of the niosomes formed, while the combined effect of the surfactant: cholesterol and lipid: drug ratio affected the EE%. In order to prepare samples with a smaller size, lower PDI, and higher EE%, the 2:1 and 20:1 molar ratios for the surfactant: cholesterol and lipid: drug were used which had a 93.95% and 96.22% EE% for both Let and Cyclo drugs with sizes of 241 nm. This NLC formulation was used to prepare NLCPFA and NL (Table 1). The results show that the EE% of the NLCPFA was higher than for the NLC samples (*p* < 0.05) for both of the drugs, and also the mean average size of the modified nanoparticles was less than the NLC with lower PDI (*p* < 0.05). Furthermore, all samples had larger size particles in comparison with NL due to the encapsulation of the hydrophobic drugs into the bilayers resulting in bilayer expansion (*p* < 0.001).

**Figure 1:**
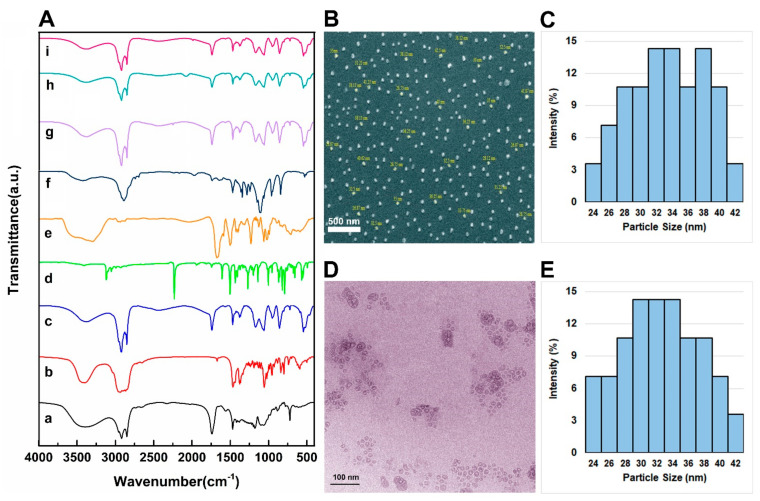
(**A**) FTIR spectra of different niosomal formulations and their component including (a) Span 60, (b) cholesterol, (c) unloaded niosomes, NL, (d) Let, (e) Cyclo, (f) folic acid, (g) niosomal Let, (h) niosomal Cyclo, and (i) NLCPFA; (**B**) FE-SEM image of NLCPFA; (**C**) average particle size and particle size distributions obtained by SEM image with an average of 33.8 nm; (**D**) TEM images of NLCPFA; (**E**) particle size and distributions by TEM images with an average of 27.9 nm.

The chemical composition of the different NL, NLC, and NLCPFA samples and their constituent components were analyzed using FTIR spectroscopy, and the results are presented in Figure 1A. In this figure, the spectra labeled from **a** to **i** are for Span 60, cholesterol, NL, Let, Cyclo, PEG, niosomal Let, niosomal Cyclo, and functionalized niosomal Let/Cyclo, respectively. The main peaks observed in these spectra are related to the functional groups of the drugs and niosome components including lipids and poly (ethylene glycol) (PEG) as presented in Table S2 [35,36,37,38,39,40]. Spectrum **g** is related to Let encapsulated into niosomes with an absorbance peak observed at 2247 cm^−1^, which can be attributed to C≡N stretching, which is damped compared to the Let alone, thus indicating successful drug encapsulation. In spectrum **h,** a peak observed at 2196 cm^−1^ was observed, which is related to O=P–OH stretching. In spectrum **i** of the NLCPFA samples, the peaks of Let and Cyclo disappeared, while a weak peak related to CH_2_ appeared. This indicated the presence of PEG and, hence, successful encapsulation. In all the structures, the spectra shifted to lower wavenumbers after the addition of the drug, indicating a change in the interaction between the drugs, cholesterol, and Span 60. This aspect confirms the drugs and cholesterol entrapment into the bilayers [41]. Furthermore, the peak at 1627 cm^−1^ of PEG in the DSPE–PEG–FA structure, related to N–H, shifted to 1604 cm^−1^ in NLCPFA, indicating a successful functionalization by PEG in the final formulation. Moreover, the morphology of the NLCPFA samples were analyzed using FE-SEM, and the results are reported in Figure 1B,C. Niosomes were fully dispersed and had an average particle size at approximately 33.8 nm. TEM images (Figure 1D) reveal bilayers of approximately 28 nm, and the size distributions are shown in Figure 1E. The contrast between the results of dynamic light scattering size and obtained mean average diameter size by electron microscopes could be the result of drying niosomes during the electronic microscope analysis process that results in measuring the exact diameter size. Furthermore, the results of the DLS show the hydrodynamic diameter of niosomes by measuring the core of nanoparticles plus adsorbed molecules such as water molecules and ions to the surface [35,42].

### 2.2. Release Study

**Figure 2:**
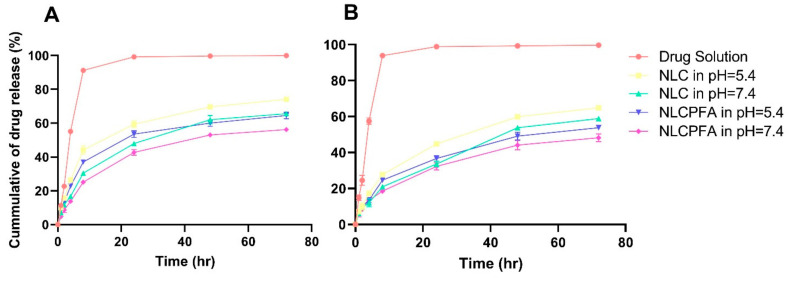
Release curves by experimental data of (**A**) Cyclo and (**B**) Let from different samples.

Release behavior of the drugs from the different niosomal formulations in physiological and acidic environments was investigated as presented in Figure 2. Drug solutions were used as a control sample, and their release was tested at pH = 7.4 in PBS (Figure 2A). The release profile from NLC and NLCPFA were investigated at pH = 5.4 and 7.4 as a cancerous and healthy environment, respectively (Figure 2B–E). As shown in these figures, drugs from the solutions have a burst release in the PBS media within the first 1 h as the drug moves across the diffusion gradient. However, the drugs encapsulated within the niosomal formulations prevent this phenomenon, where a prolonged release of both drugs was observed. The release of the drugs from niosomes was more rapid at lower pH in comparison to physiological environments (*p* < 0.05) due to the niosomes swelling and breaking or faster hydrolysis of the surfactant in the acidic conditions [43,44]. Moreover, in the same pH, slower drug release was observed from the NLCPFA because of the greater stability of the modified niosome nanoparticles. This stability comes from the greater entanglement of the folic acid molecular chains on the niosomes’ surface that protects the nanoparticles from faster hydrolysis [45]. This lower drug release observed from NLCPFA in both acidic (pH = 5.4) and physiological (pH = 7.4) conditions for both Let and Cyclo drugs in comparison with NLC. For predicting the release profile behavior, different kinetic models were used and applied to the release data of different formulations (Tables S3 and S4) [46]. The data indicates that the Korsmeyer–Peppas model has the best curve fitting with the highest R-square for both drugs in different formulations. In different pH, the release mechanism changes, and the *n* value of the model ranges in 0.46 < *n* < 0.59.

### 2.3. Cytotoxicity Assay

**Figure 3:**
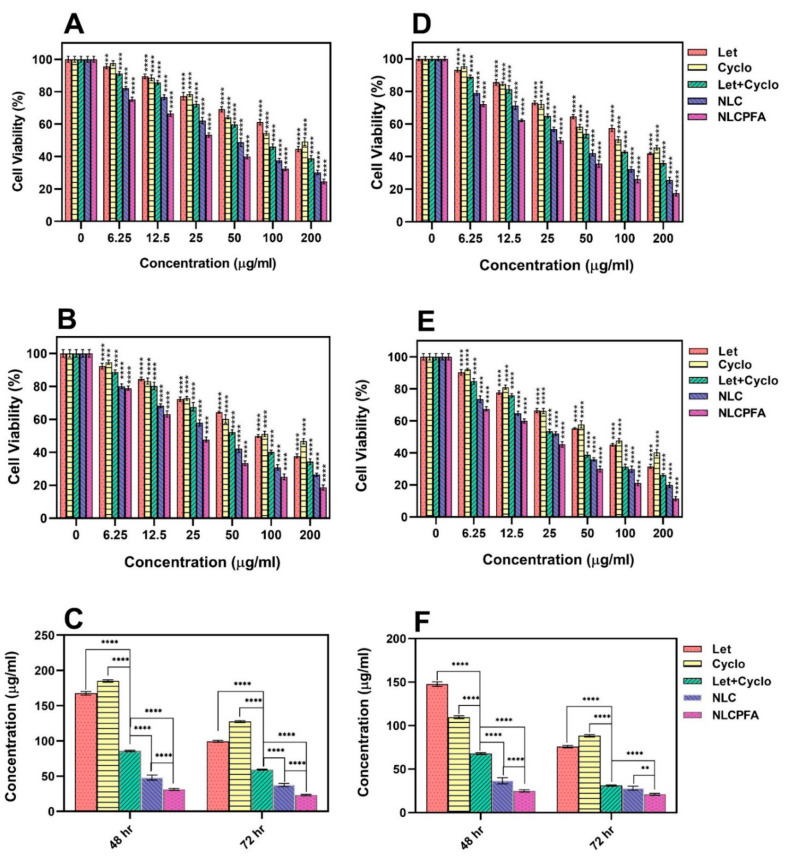
Cytotoxicity effect analysis of MDA-MB-231 (**A**–**C**) and SKBR3 (**D**–**F**) cell lines against different fabricated niosomal and non-niosomal formulation treatments in 48 h (**A**,**D**) and 72 h (**B**,**E**) by various concentration and calculated IC_50_ concentrations by linear interpolation of each sample for MDA-MB-231 (**C**) and SKBR3 (**F**) ** *p* < 0.01, and **** *p* < 0.0001).

The biocompatibility of the different niosomal formulations was tested on MCF10A cells as a healthy cell line. The results showed that the unloaded niosomes were biocompatible at the concentrations tested, where no significant effect on MCF10A viability was observed. The cytotoxicity of different samples, including Let, Cyclo, their combinations, NLC, and NLCPFA, was investigated using an MTT assay test against MDA-MB-231 and SKBR3 cells (Figure 3). Each sample was tested after 48 h and 72 h. The data indicated that both Let and Cyclo possessed cytotoxicity against both cell lines in concentrations above 12.5 µg/mL at both time points (*p* < 0.0001). In addition, a synergistic effect was observed in the data presented in the cytotoxicity results of the used Let+Cyclo data that showed that they together were more toxic against these two cell lines at any concentration and time. Moreover, the synergistic effects was calculated and quantified by measuring the combination index (CI) of each drug using median effect analysis methods presented by Chou and the results are presented in Figure S2 [47,48]. By the presented CI results that were lower than 1 in most of the used concentration, (CI < 1, CI = 1, and CI > 1 are considered as synergistic, additive, and antagonistic effects), it is concluded that these two drugs have synergistic effects in concentrations less than 125 µg/mL against MDA-MB-231 and less than 100 µg/mL against SKBR3. The cells incubated with NLC experienced a larger drop in cell viability (*p* < 0.0001), which could be due to the differing intracellular drug concentrations, as the drugs are likely to enter the cell via a different mechanism than the free drug when encapsulated inside a nanoformulation. In addition, once inside the cells, the pH-responsive nature of the niosomes, as observed in Figure 2, would ensure drug release from inside the niosomal bilayer. The NLCPFA niosomes possessed a greater cytotoxic effect on the cancerous cells; this is most likely due to the interaction of folic acid on the surface of niosomes with folate receptors on the breast cancer cells resulting in an active transport of the niosomes inside the cells [41,49]. The IC_50_ values for the free drugs and niosomes formulations are shown in Figure 3C,F. The IC_50_ values (µg/mL) for different samples were Let (167.47 ± 2.57), Cyclo (184.91 ± 1.68), Let+Cyclo (85.76 ± 1.1), NLC (47.84 ± 3.57), and NLCPFA (31.13 ± 1.35) for MDA-MB-231 cell in 48 h; Let (99.48 ± 1.42), Cyclo (127.29 ± 1.39), Let+Cyclo (59.22 ± 0.76), NLC (37.53 ± 2.33), and NLCPFA (23.18 ± 1.07) for MDA-MB-231 cell in 72 h, Let (147.71 ± 2.57), Cyclo (109.62 ± 1.68), Let+Cyclo (67.85 ± 1.1), NLC (36.67 ± 3.57), and NLCPFA (24.92 ± 1.35) for SKBR3 cell in 48 h, and Let (75.77 ± 1.42), Cyclo (88.33 ± 1.39), Let+Cyclo (31.01 ± 0.76), NLC (28.08 ± 2.33), and NLCPFA (20.94 ± 1.07) for SKBR3 cell in 72 h. In these results a synergic effect was evident when the Let and Cyclo combination was administered, which resulted in a significantly more toxic effect in comparison with each drug alone for both cell lines (*p* < 0.0001). By the obtained IC_50_ for each cell line in two time periods, the IC_50_ concentrations of Let+Cyclo together against both of cell lines in each 48 and 72 h are in the range of synergistic effects obtained by CI measurement presented in Figure S2. Moreover, an additional synergic effect was present with the formulation of NLC in contrast with using both drugs together without encapsulation (*p* < 0.0001). The NLCPFA formulations in general were more cytotoxic compared with the NLC niosomes, indicating not only that the folic acid functionalization was successful, but also confirming that active targeting had also been achieved (*p* < 0.0001).

### 2.4. Gene Expression Analysis

The caspase family possesses a cysteine protease group that executes apoptosis via receiving apoptotic signals from mitochondria as intracellular pathways of death receptors. *Caspase-9* is an initiator apoptosis member of the caspase family with a monomeric structure that contains activation domain motif activated in the multiprotein platform and completes cell break and apoptosis process [50], while *caspase-3* is a downstream effector member of the caspase family that is expressed in cancerous cells, such as MDA-MB-231 [50] and affects cell dysfunction and destruction mediated by activated *caspase-9* [51]. Also, cyclin families have a key role in proliferation and DNA synthesis in cells. For instance, retinoblastoma protein, BRCA1, and NPAT proteins phosphorylation that has an important role in cell proliferation are regulated by *cyclin-E* [51], while its overexpression has been observed in more than 25% of breast tumors. Moreover, DNA synthesis or repair is the result of the regulation of the *cyclin-D* overexpression that causes gene promotion. Additionally, MMP families augment cancerous cell activities, such as proliferation, inflammation, invasion, and especially angiogenesis, by degrading the extracellular matrix [52]. Furthermore, it is reported that the degrees of *MMP-9* expression were directly associated with NF-kB activity in cancerous prostate PC-3 cells. By the results, upregulation in the caspase family and downregulation in cyclin and MMP family result in more apoptosis rate in breast tumors.

**Figure 4:**
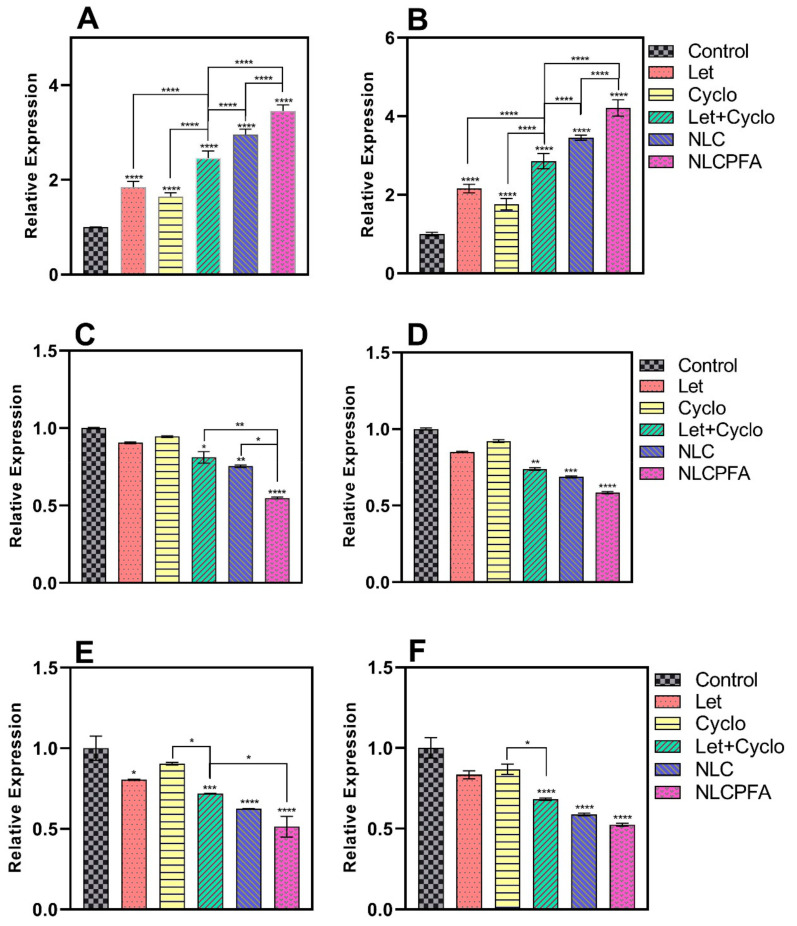
The expression level changes of (**A**) *caspase-3*; (**B**) *caspase-9*; (**C**) *cyclin-D*; (**D**) *cyclin-E*; (**E**) *MMP-2*; (**F**) *MMP-9* genes in MDA-MB-231 cancerous cell line after 72 h treatment by co-loading Let and Cyclo in different niosomal formulations at their IC_50_ concentration (* *p* < 0.05, ** *p* < 0.01, *** *p* < 0.001, and **** *p* < 0.0001).

**Figure 5:**
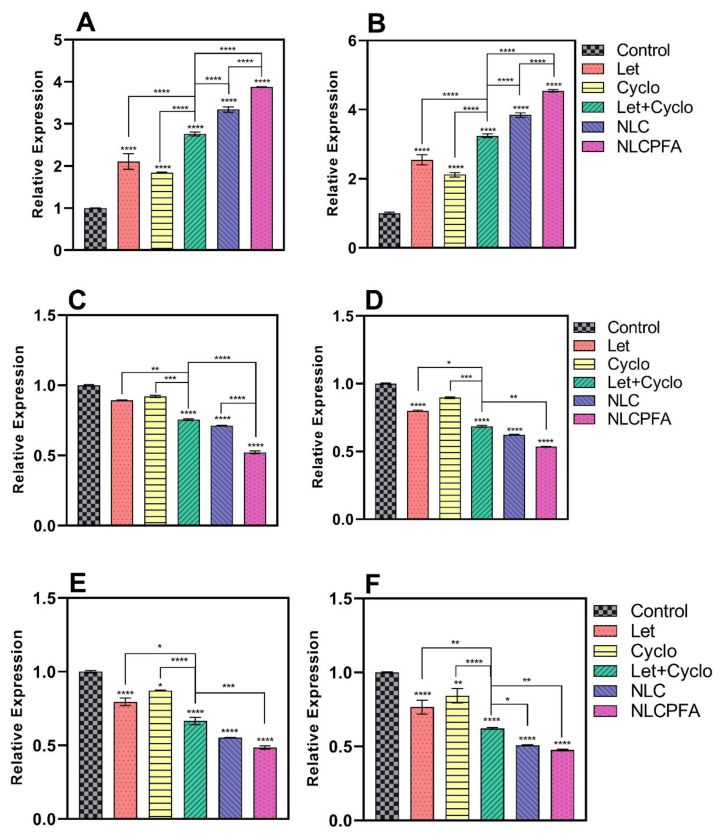
The expression level changes of (**A**) *caspase-3*; (**B**) *caspase-9*; (**C**) *cyclin-D*; (**D**) *cyclin-E*; (**E**) *MMP-2*; (**F**) *MMP-9* genes in SKBR3 cancerous cell line after 72 h treatment by co-loading Let and Cyclo in different niosomal formulations at their IC_50_ concentration (* *p* < 0.05, ** *p* < 0.01, *** *p* < 0.001, and **** *p* < 0.0001).

Chemotherapy drugs can have various effects on different genes expression levels in two main pro-apoptotic and anti-apoptotic gene groups. For instance, Let and Cyclo treatments can lead to different gene expression levels in breast cancer cells [52,53,54]. Therefore, *caspase-3*, *caspase-9*, *cyclin-D*, *cyclin-E*, *MMP-2,* and *MMP-9* genes were considered to measure gene expression levels in MDA-MB-231 and SKBR3 breast cancer cells. We used six groups to evaluate the effects on gene expression, which included a control group, conventional formulation of Let, conventional formulation of Cyclo, combination of conventional formulations of Let and Cyclo, niosomes co-loaded with Let and Cyclo (NLC), and functionalized niosomes co-loaded with Let and Cyclo (NLCPFA). Figure 4 and Figure 5 illustrate that an upregulation was observed in *caspase-3* and *caspase-9* gene expression levels, whereas a downregulation was seen in *cyclin-D*, *cyclin-E*, *MMsP-2,* and *MMP-9* expression levels in treatment groups compared with the control group. In almost all cases the conventional formulations Let and Cyclo—when used alone—showed a reduction MMP and cyclin gene expression (*p* < 0.05 or *p* < 0.01) and a significant increase in caspase genes expression (*p* < 0.0001) in comparison with the control group. Moreover, synergistic effects were observed with the combination of the conventional formulation of Let and Cyclo, where the treatment resulted in a lower expression of each MMP and cyclin genes (*p* < 0.001) and higher expression of caspase genes (*p* < 0.0001) in comparison with the conventional formulation of both of the drugs when used alone. Furthermore, the NLC group caused a higher upregulation in caspase genes and a smaller downregulation in each MMP and cyclin genes in comparison with conventional formulation of Let and Cyclo (*p* < 0.01 or *p* < 0.001). Finally, NLCPFA treatment led to a significantly higher increase in *caspase-3* and *Caspase-9* and a more significant decrease in *cyclin-D*, *Cyclin-E*, *MMP-2,* and *MMP-9* expression levels compared with the NLC group (*p* < 0.001 or *p* < 0.0001). These results could be due to the fact that there was a higher cellular uptake when functionalized niosomes were used compared to non-functionalized niosomes.

### 2.5. Apoptosis Analysis

**Figure 6:**
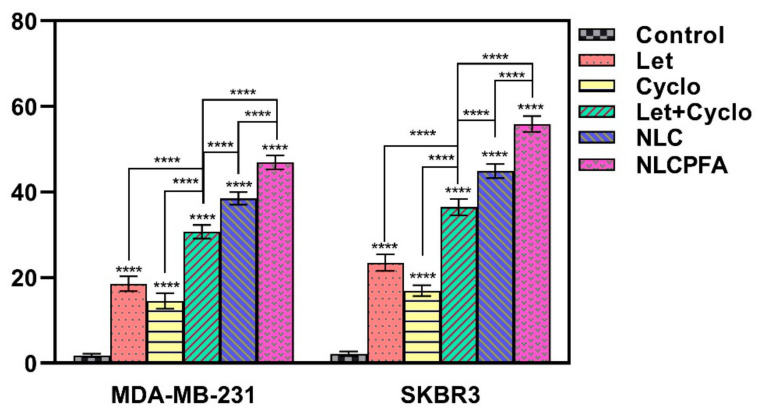
Total apoptosis analysis of MDA-MB-231 and SKBR3 cancer cell lines after treating by free drugs and different niosomal formulations presenting Q2+Q3, where the Q2 and Q3 quadrants represent late and early apoptosis stained by FITC^+^/PI^+^ and FITC^+^/PI^‒^, respectively (**** *p* < 0.0001).

Different stages of apoptosis or programmable cell death can be detected using flow cytometry by staining with special colored apoptosis proteins. In the initial steps of apoptosis, phosphatidylserine was released from the cytosol to the axoplasmic cellular membrane, and annexin V, a protein with a high ability to bind phosphatidylserine, reacted with that. The initial apoptosis steps will be detected using flow cytometry after conjugation of annexin V with fluorescein isothiocyanate (FITC). In addition, in other apoptosis steps, cellular membrane penetration changes, and some staining colors can permeate into the cells while these colors are not diffusible in live cells. Thus, live and dead cells are detectable by DNA staining using PI in the final apoptosis steps. Finally, the use of different staining colors and differentiating apoptotic, necrotic, death, and healthy cells can help apoptosis steps classification. For measuring different apoptosis steps in cancerous cells, MDA-MB-231 and SKBR3 cancer cells lines were treated by different niosomal formulations in their IC_50_ concentrations for 48 h and then double-stained by FITC and PI using flow cytometry. The results are presented in Figure 6 that shows Q2+Q3 as total apoptosis death. By the results, each drug cause much more apoptotic death in both cell lines in comparison with cancerous cells without treatment as control groups (*p* < 0.0001). Moreover, Let and Cyclo have synergic effects in total apoptosis of cancerous cells by 65.6% and 111.2% apoptosis increment in MDA-MB-231 and 55.3% and 115% ones in SKBR3 cells in comparison with each Let and Cyclo drugs alone, respectively (*p* < 0.0001). The results revealed that NLC enhances apoptosis’ effects in 25.2% and 23.1% increments in total apoptotic death in MDA-MB-231 and SKBR3, respectively, in comparison with using both Let and Cyclo drugs together without niosomal encapsulation (*p* < 0.0001). In addition, NLCPFA increases total apoptosis in treated cancer cell lines significantly in 21.9% and 24.5% increment in MDA-MB-231 and SKBR3, respectively in contrast with NLC samples (*p* < 0.0001). As a consequence, all of these results confirm the other’s toxicity and gene expression results and agree with them.

### 2.6. Cell Cycle Analysis

**Figure 7:**
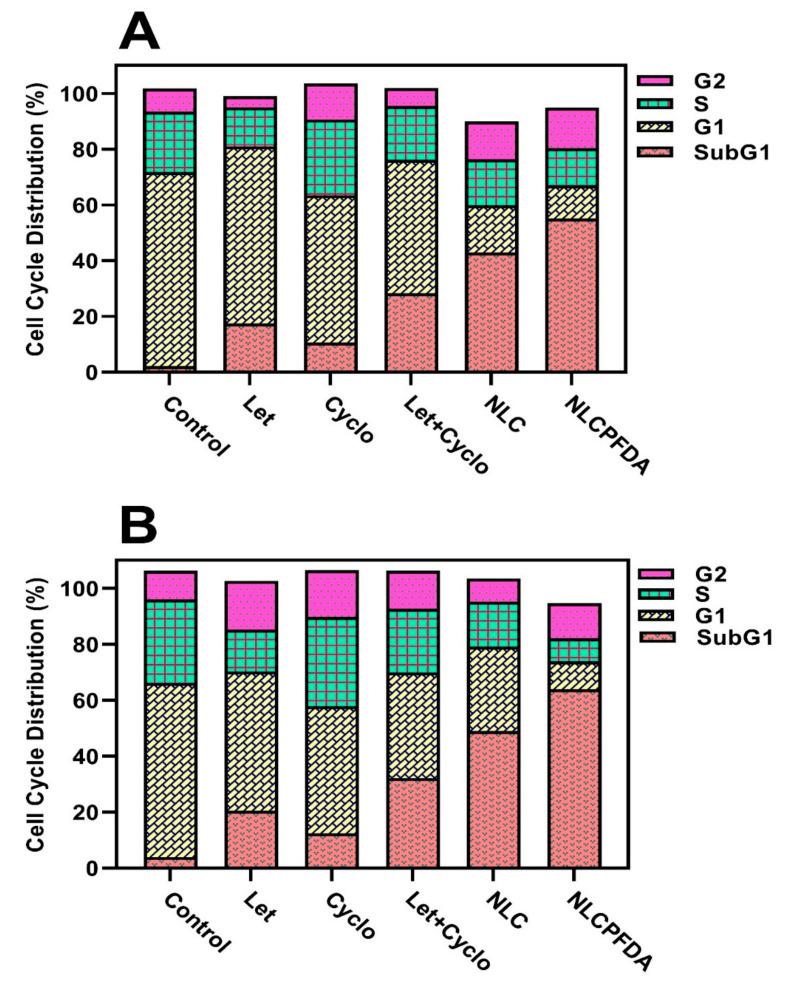
Cell cycle distribution for (**A**) MDA-MB-231 and (**B**) SKBR3 cancerous cell lines after treating by different niosomal formulations and free drugs.

The effect of the drugs and niosomal formulations on the MDA-MB-231 and SKBR3 cell cycle was investigated using a flow cytometry test and the results are presented in Figure 7. The results demonstrate a synergic effect and niosomal encapsulation of both drugs causes shifting toward sub-G1 phase in both of MDA-MB-231 and SKBR3 breast cancer cells. Furthermore, NLCPFA produces a greater shift demonstrating more drug efficiency. These results showed a 51.8% and 95.1% increment when shifting to a sub-G1 phase in MDA-MB-231 and 52.2% and 98.6% in the SKBR3 cell lines for NLC and NLCPFA, respectively, compared to free drugs that confirmed the increase in the apoptosis as observed and discussed in previous sections.

## 3. Materials and Methods

### 3.1. Materials

Niosome fabrication reagents, including cholesterol, Span 60, chloroform, and 1,2-distearoyl-sn-glycero-3phosphoethanolamine-N-(folate (polyethylene glycol)-2000) (ammonium salt) (DSPE-PEG (2000)-Folate), were purchased from Merck (Munich, Germany). Let and Cyclo were purchased from Bio Basic (Markham, ON, Canada). Roswell Park Memorial Institute (RPMI-1640), fetal bovine serum (FBS), penicillin–streptomycin, phosphate-buffered saline (PBS), and trypsin–EDTA as cell culture reagents were purchased from Gibco (Carlsbad, CA, USA). Dimethyl sulfoxide (DMSO) and 3-(4, 5-dimethylthiaol-2-yl)-2, 5-diphenyltetrazolium bromide (MTT) were purchased from Sigma–Aldrich (Munich, Germany). MDA-MB-231 and SKBR3 cell lines were obtained from the Pasteur Institute of Iran (Tehran, Iran). An RNA extraction kit was procured from Qiagen (Germantown, MD, USA). A RevertAid First Strand cDNA Synthesis Kit (Fermentas, Vilnius, Lithuania) was used to synthesize the cDNA.

### 3.2. Fabrication of Nanoniosomes

**Scheme 1:**
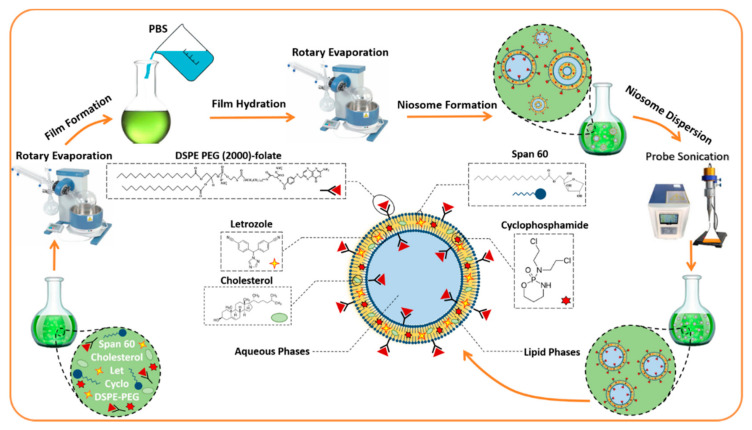
The potential structure of the Let and Cyclo co-loaded in PEG functionalized niosomal formulation by the schematic of a bilayer niosome.

A thin-layer film hydration method was used to prepare unloaded (NL), drug-loaded niosomes (NLC), and functionalized drug-loaded niosomes (NLCPFA). Firstly, lipids containing Span 60 and cholesterol were dissolved in 10 mL of chloroform and, subsequently, the solvent was removed via rotary evaporation at 160 rpm, 60 °C for 30 min, forming a dried thin-layer film residue. The films were hydrated with 10 mL of phosphate buffered saline (PBS) using a rotary evaporator at 150 rpm, 25 °C for 30 min. The samples were sonicated for 5 min to obtain a uniform size distribution, and, finally, they were stored at 4 °C until further use. NLC and NLCPFA nanoparticles were prepared as above, but the drugs (10 mg of Let and 10 mg of Cyclo) and DSPE-PEG (2000)-Folate (0.02% mmol) were added to lipids in the solvation step. Drugs contained Let and Cyclo in all nanoparticle samples, and just for synergetic studies, they were used alone in some cell study cases. Lipid:drug and surfactant:cholesterol molar ratios were investigated to optimize physical and chemical parameters such as size, polydispersity index (PDI), and entrapment efficiency. Defined parameters were used for preparing functionalized niosomes in an optimum molar ratio of components and compared by ordinary ones as the functionalization effect. A schematic of the NLCPFA nanoparticle is presented in Scheme 1, showing entrapment of Let and Cyclo in the lipid phase between bilayers as two hydrophobic drugs.

### 3.3. Entrapment Efficiency

The number of entrapped drugs in the nanoparticles was measured via an ultrafiltration system using a PBS/sodium dodecyl sulfate (SDS) solution, 30 kDa Ultra-15-membrane, centrifugation at 4000× *g* for 20 min. In this system, drug-loaded niosomes remained at the top of the chamber and free unloaded drugs passed through the filter residing in the bottom portion of the chamber. The concentration of free drug in the passed solution were measured using UV–Visible spectroscopy after comparison to a calibration of known standards (*R*^2^ = 0.99). Entrapment efficiency (EE%) was calculated using the following equation:

(1) $$\mathrm{EE}(\%)=\frac{A-B}{A}\;\times \;100$$

where A and B are the numbers of drugs loaded into the formulation and passed through the filter drug, respectively.

### 3.4. Chemical Structure Analysis

The chemical composition of the different samples, such as niosome, their constituent components, including Span 60 and cholesterol, unloaded NL, different used drugs, including Let and Cyclo, folic acid, niosomal Let, niosomal Cyclo, and NLCPFA, were analyzed using Fourier transform infrared (FTIR) spectroscopy (Spectrum Two, Waltham, MA, USA). All the samples were lyophilized, and their powder was mixed into KBr and placed in front of the beam by pellets and analyzed by a range of 400–4000 cm^−1^ at 25 °C.

### 3.5. Size, Polydispersity, and Morphological Investigations

The particle size and PDI of different formulations were analyzed using a Malvern Nanosizer Zeta DS (Malvern Instrument Ltd., Malvern, UK). All the samples were analyzed after 10 times dilution in water at room temperature. Transmission electron microscopy (TEM) and field emission scanning electron microscopy (FE-SEM) was used to image the nanoparticles. For FE-SEM analysis, a drop of the sample was imaged on a NOVA NANOSEM, 450 FEI by 15 kV in voltage was used in a vacuum after coating a 100 angstrom layer of gold by PVD coating in 3 min under argon at 0.2 atm pressure.

### 3.6. Drug Release Study

The release of drugs from NLC and NLCPFA was investigated using in vitro dialysis. Firstly, 2 mL of each sample was pipetted into a 12 kDa dialysis membrane and placed into 50 mL of PBS-SDS (0.5% *w*/*v*) solution at pH = 5.4 and 7.4 at 37 °C with stirring. Each sample was put into the dialysis bag separately and also sunk in a separated PBS solution. Dialysis bags containing non-capsulated and capsulated drug were as supplier phase and the PBS solutions were as receiver phase. At predetermined time intervals, 1 mL of each release medium was withdrawn and replaced with an equal volume of fresh solution. Drug concentration was quantified using UV/Visible spectroscopy (JASCO, V-530, Japan) at 260 nm for Let (*R*^2^ = 0.996) and 210 nm for Cyclo (*R*^2^ = 0.992), respectively. Drug release profiles were plotted for different samples and also analyzed by different kinetic models. Furthermore, some samples containing only drug solutions in the same initial concentration as formulations and other dialysis conditions were used as control.

### 3.7. Cell Culture

In vitro studies investigated using two different breast cancer cell lines: human breast adenocarcinoma cell lines (MDA-MB-231 and SKBR3). These two cell lines were cultured in RPMI-1640 media supplemented with 10% of fetal bovine serum (FBS) and 1% of penicillin–streptomycin in a 5% CO_2_ and 37 °C atmosphere. Cells were passaged every 5–6 days by removing the old media, washing with PBS, incubating within 2–3 mL of trypsin/EDTA for cell detachment, subsequently after centrifugation, the cell pellet was transited into two flasks in fresh media.

### 3.8. Cytotoxicity Assay

Cytotoxicity of different samples was determined using the 3-(4,5-dimethylthiazol-2-yl)-2,5-diphenyl-2H-tetrazolium bromide (MTT) assay. MDA-MB-231 and SKBR3 were seeded to 96-well plates, 10,000 cells per well. Next, different samples such as Let, Cyclo, Let+Cyclo, NLC, and NLCPFA were added to the media in different concentrations and incubated with the cells for two days. After this, MTT solution (0.5 mg/mL) was supplemented to the treated cells. The cells were incubated for 2 h, after which, the MTT solution of each well was removed and replaced with DMSO (100 μL) to reduction of the colorless tetrazolium dye MTT to insoluble formazan, which has a purple color. The absorbance of each well was measured at 570 nm using a plate reader and compared with control samples. The concentration related to the half-maximal inhibitory concentration (IC_50_) of different samples were calculated, all other cellular analyses were performed above IC_50_ concentrations.

### 3.9. Gene Expression Analysis

Real-time polymerase chain reaction (PCR) was used to evaluate the apoptotic gene expression in the cell lines. Cells were cultured and treated with different samples, and then their RNA was extracted using an RNA extraction kit (Qiagen, Germantown, MD, USA) following the instructions in the assay kit. A photonanometer (IMPLEN GmbH, München, Germany) was used for measuring the concentration of extracted RNA. Then, a Revert AidTM First Strand cDNA Synthesis Kit (Fermentas, Vilnius, Lithuania) was used for synthesizing cDNA by a mixture of reaction components including reaction buffer (5 µL), extracted RNA (1 µg), random hexamer primer (0.5 µL), oligo dT primer (0.5 µL), a mixture of deoxynucleotide triphosphate (2 µL), RNase enzyme inhibitor (1 µL), reverse transcript enzyme (1 µL) and distilled water (up to 20 µL) and temperature program set to 25 °C for 5 min as primer annealing, 42 °C for 1 h, 70 °C for 5 min and 4 °C for 5 min. Finally, the real-time PCR reactions were performed at 95 °C for 1 min, 95 °C for 15 s, and 60 °C for 1 min by a light cycler (Bioneer, Daejeon, South Korea). Relative gene expression was calculated using the ΔΔCt method by assuming 100% efficiency. *Caspase-3*, *caspase-9*, *cyclin-D*, *cyclin-E*, *MMP-2*, and *MMP-9* were chosen as target genes, and their primer sequences were prepared as following (Table S1).

### 3.10. Flow Cytometry

The apoptosis/necrosis ratio was evaluated on MDA-MB-231 and SKBR3 cells. The cells (100,000) were seeded into the plate and treated with different drug samples at their respective IC_50_ concentration. The cell samples analyzed using the Annexin V/propidium iodide (PI) assay after 48 h incubation, using an apoptosis detection kit (Roch, Mannheim, Germany). Untreated samples were used as a control. Finally, flow cytometry was used for evaluating apoptosis and necrotic level.

### 3.11. Cell Cycle Analysis

MDA-MB-231 and SKBR3 cells (1,000,000 per well) were seeded into 6-well plates and incubated in media for 24 h. After this time, the cells were treated with different drug samples at their respective IC_50_ concentration for 48 h. Next, cell samples were detached and fixed using 70% ethanol at 4 °C for 24 h. Finally, samples were stained with 500 µL of a PI solution containing RNase in darkness for 20 min at 25 °C and analyzed using flow cytometry.

### 3.12. Statistical Analysis

Statistical analysis and curve fitting were performed using GraphPad Prism software version 8 (GraphPad Software, Inc., San Diego, CA, USA). Data from three independent experiments were expressed as means ± standard deviations. Statistical significance was determined with a one-way analysis of variance, after validating the normality and homoscedasticity of the data sets. For all analyses, statistical significance was pre-set at α = 0.05.

## 4. Conclusions

For enhancing the drug delivery system as a new treatment method for breast cancer therapy, niosome nanoparticles as a nanocarrier was fabricated and then letrozole and cyclophosphamide, as commonly used chemotherapies for breast cancer with differing mechanisms of action, were co-loaded into them. Niosomes were fabricated by the thin-layer film method using cholesterol and Span 60 as surfactant. Optimizing formulation characteristics were investigated using different surfactant-to-cholesterol molar ratios of 1:1 and 2:1 and lipid-to-drugs molar ratios of 10:1 and 20:1. The molar ratios of 2:1 and 20:1 molar ratios, respectively, were selected for surfactant to cholesterol and lipid to the drug by the lowest nanoparticle size and highest drug entrapment efficiency results. The drug release behavior of the nanoparticles showed unburst pH-dependent controllable release with lower release at pH = 7.4 and a higher one at pH = 5.4 at physiological and cancerous situations, respectively. In addition, the MTT assay showed considerable cytotoxicity of co-loaded niosomal formulations against MDA-MB-231 and SKBR3 cancer cell lines in particular in the case of NLCPFA formulation. The in vitro studies showed significant apoptosis in co-loaded niosome encapsulation and even much more in functionalized niosome nanoparticles by upregulation in the expression levels of *caspase-3* and c*aspase-9* as pro-apoptotic genes and downregulating *cyclin-D*, *cyclin-E*, *MMP-2,* and *MMP-9* as genes contributing to cancer development and progression. By the obtained results, it could be concluded that co-loading both letrozole and cyclophosphamide in fabricated niosome nanoparticles by optimum formulation and functionalizing by folic acid would be suitable and reliable for use in breast cancer therapy.

## Figures and Tables

**Table 1:** Different formulations used for niosomes preparation with their physical properties including size, PDI, and EE%. In this chart, NL, NLC, and NLCPFA are unloaded, drug-loaded, and functionalized drug-loaded niosomes, respectively.

Sample	Surfactant: Cholesterol (Molar Ratio)	Lipid: Drug (Molar Ratio)	Let (mg)	Cyclo (mg)	Size (nm)	PDI	EE (%)	
							Cyclo	Let
NLC	1:1	10:1	10	10	224.7 ± 6.4	0.224 ± 0.009	89.60 ± 0.86	91.58 ± 0.96
NLC	2:1	10:1	10	10	231.3 ± 7.2	0.257 ± 0.011	92.75 ± 1.06	93.50 ± 1.12
NLC	1:1	20:1	10	10	251.7 ± 5.3	0.249 ± 0.010	91.39 ± 0.98	94.85 ± 1.23
NLC	2:1	20:1	10	10	240.9 ± 4.4	0.198 ± 0.008	93.95 ± 1.36	96.22 ± 1.42
NLCPFA	2:1	20:1	10	10	213.9 ± 3.2	0.143 ± 0.007	94.10 ± 1.85	98.50 ± 1.88
NL	2:1	20:1	-	-	186.1 ± 3.1	0.158 ± 0.009	-	-

## Data Availability

Data are contained within the article and Supplementary Material.

## Supplementary Materials

The following are available online at https://www.mdpi.com/article/10.3390/ph15010006/s1, Table S1: Sequence of primers related to different genes in forward and revers form used in Real-time PCR; Table S2: The main characteristic peaks of FTIR spectra related to different niosomal formulations and their components; Table S3: Different kinetics models for Let released from different niosomal formulations in physiological and acidic conditions at 37 °C; Table S4: Different kinetics models for Cyclo released from different niosomal formulations in physiological and acidic conditions at 37 °C; Figure S1: Particle size distributions of (**a**) NL, (**b**) NLC with optimum parameters, and (**c**) NLCPFA formulations prepared by DLS analysis. Figure S2: Combination Index (CI ) of Let and Cyclo drug used in niosome nanoparticles against (**A**) MDA-MB-231 after 48 h, (**B**) MDA-MB-231 after 72 h, (**C**) SKBR3 after 48 h, and (**D**) SKBR3 after 72 h.
