# Supplementary material for: Co-Delivery of Letrozole and Cyclophosphamide via Folic Acid-Decorated Nanoniosomes for Breast Cancer Therapy: Synergic Effect, Augmentation of Cytotoxicity, and Apoptosis Gene Expression
Source: Pharmaceuticals (Basel). 2021 Dec 21;15(1):6. doi: 10.3390/ph15010006 (PMC8780158; doi:10.3390/ph15010006)
Supplement: Supplementary file 1 [file pharmaceuticals-15-00006-s001.zip › pharmaceuticals-1499475-supplementary.pdf]

Table S1. Sequence of primers related to different genes in forward and revers form used in Real-time PCR.

Gene	Sequence of primers
Caspase-3	Forward: 5'- CATACTCCACAGCACCTGGTTA-3′
	Revers: 5'- ACTCAAATTCTGTTGCCACCTT-3'
Caspase-9	Forward: 5′-CATATGATCGAGGACATCCAG-3
	Revers: 5′-TTAGTTCGCAGAAACGAAGC-3′
Cyclin-D	Forward: 5'- CAGATCATCCGCAAACACGC-3′
	Revers: 5'- AAGTTGTTGGGGCTCCTCAG-3'
Cyclin-E	Forward: 5'- CTCCAGGAAGAGGAAGGCAA-3′
	Revers: 5'- TTGGGTAAACCCGGTCATCA-3'
MMP-2	Forward: 5′- F: TTG ACG GTA AGG ACGGAC TC-3′
	Revers: 5′- CAT ACT TCA CAC GGA CCA CTTG -3′
MMP-9	Forward: 5'- GCACGACGTCTTCCAGTACC -3′
	Revers: 5'- CAGGATGTCATAGGTCACGTAGC -3'
b-actin	Forward: 5′- TCCTCCTGAGCGCAAGTAC-3′
	Revers: 5′- CCTGCTTGCTGATCCACATCT-3′

Table S2. The main characteristic peaks of FTIR spectra related to different niosomal formulations and their components.

Sample, Chemicals	Peak cm^{-1}	Description
**Span 60**	1125	C-O Stretching
	2800-3000	C-H Stretching
	3452	OH Stretching
**Cholesterol**	1747	C=O Stretching
	2800-3000	C-H Stretching
	3452	OH Stretching
	1035-1378	CH2 bending and deformation
	1506	C-C Stretching of the aromatic ring
	1674	C=C Stretching
**Niosome**	1125	C-O Stretching
	1747	C=O Stretching
	2800-3000	C-H Stretching
	3452	OH Stretching
**Let**	2229	C≡N Stretching
	3116	Sp2 C-H Stretching
	690-900	Out of plane C-H bending
	1263	C-N Stretching
**Cyclo**	3400	N-H Stretching (weak)
	1100	N-H Stretching (medium)
	1985	P=O Stretching
	1300	$-CH_2-Cl$
	950	C-N
	2370	O=P-OH (singular-strong)
**DSPE-PEG-FA**	3325	Benzene ring in FA
	1693	-C=O
	2927	-NH-
	1625, 2850	O=C-NH
	1468, 1564	C=C Stretching vibration ring in FA
	1178	P=O Stretching vibration in DSPE
	1643, 1736	C=O in DSPE

	3374	OH in the ring of FA
	2851, 2919	C-H Stretching in the aliphatic structure of PEG

Table S3. Different kinetics models for Let released from different niosomal formulations in physiological and acidic conditions at 37°C.

Release Model	Equation	R^2, n				
		**NLC-L (pH=7.4)**	**NLC-L (pH=5.4)**	**NLCPFA -L (pH=7.4)**	**NLCPFA -L (pH=5.4)**	**Let solution (pH=7.4)**
Zero-Order	$C_t=C_0+K_0t$	R^2=0.9406	R^2=0.8892	R^2=0.9110	R^2=0.8881	R^2=0.4764
Korsmeyer-Peppas	$M_t/M_\infty=K_t^n$	R^2=0.9935	R^2=0.9830	R^2=0.9927	R^2=0.9800	R^2=0.7877
		n=0.5401	n=0.5323	n=0.5059	n=0.5119	n=0.4283
First-Order	$LogC=LogC_0+K_t/2.303$	R^2=0.9701	R^2=0.9469	R^2=0.9438	R^2=0.9341	R^2=0.8060
Higuchi	$Q=K_H\sqrt{t}$	R^2=0.9902	R^2=0.9787	R^2=0.9876	R^2=0.9768	R^2=0.6484

Table S4. Different kinetics models for Cyclo released from different niosomal formulations in physiological and acidic conditions at 37°C.

Release Model	Equation	R^2, n				
		**NLC-C (pH=7.4)**	**NLC-C (pH=5.4)**	**NLCPFA -C (pH=7.4)**	**NLCPFA-C (pH=5.4)**	**Cyclo solution (pH=7.4)**
Zero-Order	$C_t=C_0+K_0t$	R^2=0.8620	R^2=0.7860	R^2=0.8483	R^2=0.7804	R^2=0.4944
Korsmeyer-Peppas	$M_t/M_\infty=K_t^n$	R^2=0.9762	R^2=0.9418	R^2=0.9676	R^2=0.9420	R^2=0.7860
		n=0.5289	n=0.4670	n=0.5811	n=0.4799	n=0.4765
First-Order	$LogC=LogC_0+K_t/2.303$	R^2=0.9247	R^2=0.8907	R^2=0.8995	R^2=0.8585	R^2=0.9190
Higuchi	$Q=K_H\sqrt{t}$	R^2=0.9648	R^2=0.9172	R^2=0.9580	R^2=0.9147	R^2=0.6679

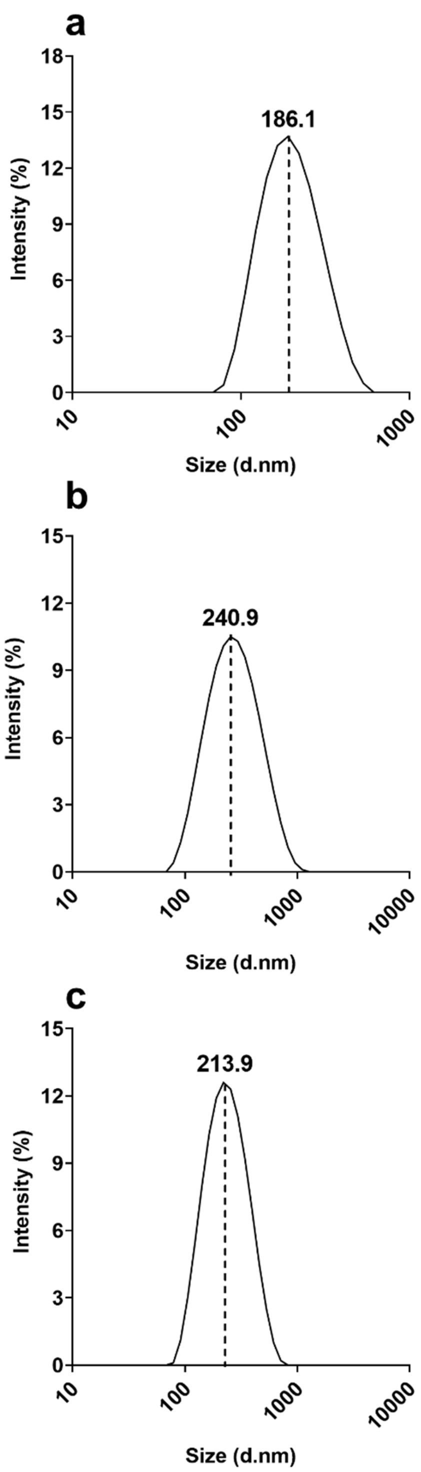

Figure S1. Particle size distributions of a) NL, b) NLC with optimum parameters, and c) NLCPFA formulations prepared by DLS analysis.

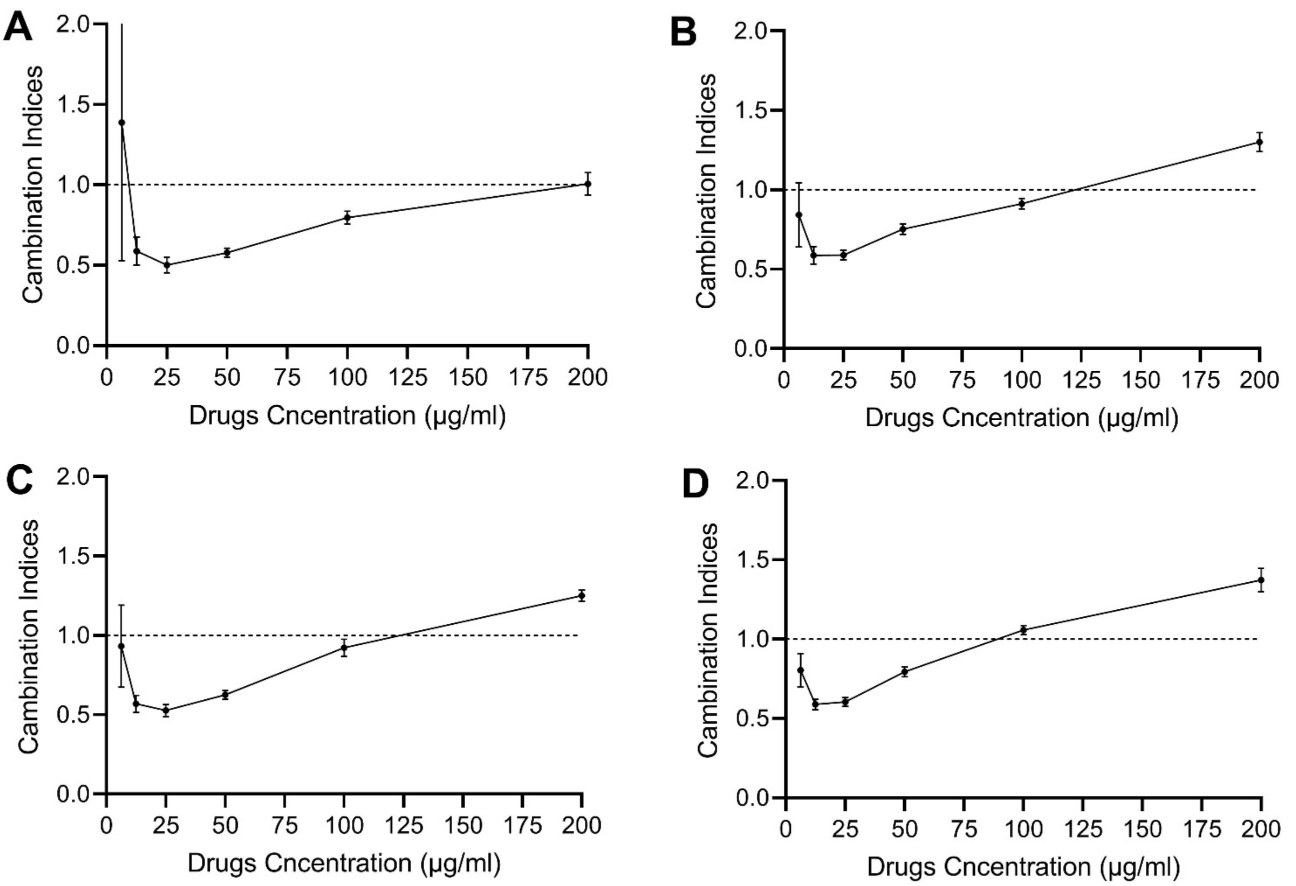

Figure S2. Combination Index (CI) of Let and Cyclo drug used in niosome nanoparticles against A) MDA-MB-231 after 48h, B) MDA-MB-231 after 72h, C) SKBR3 after 48 h, and D) SKBR3 after 72h.
